# Brain-machine interface cursor position only weakly affects monkey and human motor cortical activity in the absence of arm movements

**DOI:** 10.1038/s41598-018-34711-1

**Published:** 2018-11-05

**Authors:** Sergey D. Stavisky, Jonathan C. Kao, Paul Nuyujukian, Chethan Pandarinath, Christine Blabe, Stephen I. Ryu, Leigh R. Hochberg, Jaimie M. Henderson, Krishna V. Shenoy

**Affiliations:** 1 Neurosurgery Department, Stanford University, Stanford, CA USA; 2 Electrical Engineering Department, Stanford University, Stanford, CA USA; 3 Bioengineering Department, Stanford University, Stanford, CA USA; 4 Stanford Wu Tsai Neurosciences Institute, Stanford University, Stanford, CA USA; 5 Bio-X Program, Stanford University, Stanford, CA USA; 6 Neurobiology Department, Stanford University, Stanford, CA USA; 7 Howard Hughes Medical Institute at Stanford University, Stanford, CA USA; 8 Neurosurgery Department, Palo Alto Medical Foundation, Palo Alto, CA USA; 9 Center for Neurorestoration and Neurotechnology, Rehabilitation R&D Service, VA Medical Center, Providence, RI USA; 10 School of Engineering and Carney Institute for Brain Science Brown University, Providence, RI USA; 11 Department of Neurology, Harvard Medical School, Boston, MA USA; 12 Center for Neurotechnology and Neurorecovery, Department of Neurology, Massachusetts General Hospital, Boston, MA USA; 13 Electrical and Computer Engineering Department, University of California at Los Angeles, Los Angeles, CA USA

## Abstract

Brain-machine interfaces (BMIs) that decode movement intentions should ignore neural modulation sources distinct from the intended command. However, neurophysiology and control theory suggest that motor cortex reflects the motor effector’s position, which could be a nuisance variable. We investigated motor cortical correlates of BMI cursor position with or without concurrent arm movement. We show in two monkeys that subtracting away estimated neural correlates of position improves online BMI performance only if the animals were allowed to move their arm. To understand why, we compared the neural variance attributable to cursor position when the same task was performed using arm reaching, versus arms-restrained BMI use. Firing rates correlated with both BMI cursor and hand positions, but hand positional effects were greater. To examine whether BMI position influences decoding in people with paralysis, we analyzed data from two intracortical BMI clinical trial participants and performed an online decoder comparison in one participant. We found only small motor cortical correlates, which did not affect performance. These results suggest that arm movement and proprioception are the major contributors to position-related motor cortical correlates. Cursor position visual feedback is therefore unlikely to affect the performance of BMI-driven prosthetic systems being developed for people with paralysis.

## Introduction

BMIs are an emerging medical technology that can be used to bypass motor disabilities due to injury and disease. These systems read out the movement intentions of people with paralysis to restore function^1^, for example by controlling computer cursors for communication^2^, commanding arm and hand movements of robotic limbs^3^, or electrically stimulating the person’s own paralyzed muscles^4^. A key component of BMI systems is the decoder, which attempts to infer the user’s movement intentions from recorded neural activity^5^. One challenge for decoders is that neural signals in areas like motor cortex reflect other processes besides just the user’s intended movement. Motor control requires an accurate estimate of the position of the limb^6^. Such state estimation, which is mediated by both visual and proprioceptive feedback^7^ as well as efference copy^8^, adds additional sources of motor cortical modulation that may interfere with decoding movement intentions.

In particular, it has been shown that activity in primary motor (M1) and dorsal premotor cortex (PMd), which we will refer to together as ‘motor cortex’, covaries with the arm’s position in the workspace during static hold^9–12^ and during movement^10,11,13^. Activity also differs when movements in a similar direction are made with different arm orientations^14,15^. Positional effects could reflect a number of causes beyond just differences in the muscle forces that are needed to move or maintain the arm’s position: these include changes in the internal state estimate of where the arm is in space, or different proprioceptive and visual feedback. It has been hypothesized that similar effector state-related information will be present during BMI use^16–19^, and BMI performance was shown to suffer when there was a mismatch between proprioceptive and visual feedback, indicating the presence of sensory feedback from both modalities^20^. Motor cortical modulation due to effector state estimation and visual feedback is particularly germane to BMI decoding, since these factors would presumably still affect the observed neural activity even when BMI output commands should be the same.

The ReFIT decoder^19^, a state-of-the-art BMI decoder^21–23^, incorporates a ‘Cursor Position Subtraction’ operation that attempts to mitigate the anticipated neural variability due to the cursor’s position. Cursor Position Subtraction models the neural activity due to the on-screen cursor position and subtracts it out from the measured neural activity prior to decoding cursor velocity commands. Gilja, Nuyujukian and colleagues showed that this operation improves performance in monkeys, but this test was performed during BMI use accompanied by arm reaching^19^. During this behavioral context, motor cortex is expected to receive strong proprioceptive inputs and contribute efferent commands to the arm. The underlying assumption of whether there are substantial cursor positional effects during BMI use in the absence of overt movement – which is likely to be the situation for BMI users with paralysis or amputation – has not previously been tested. Indeed, when ReFIT was successfully translated to clinical study human BMI users^2,24^, the Cursor Position Subtraction operation led to mixed results^24^.

We investigated this question using a combination of pre-clinical monkey experiments and data from the BrainGate2 BMI clinical trial. We first present closed-loop decoder comparisons which show that Cursor Position Subtraction only improves performance when monkeys also made accompanying arm movements. To better understand why this was the case, we present the results of additional monkey experiments that compare cursor positional effects when the same task was performed either with the hand or with a BMI in the absence of overt movements. To tease apart whether hand and cursor position both separately influence motor cortical activity during free-arm BMI use, we analyzed further experiments intended to reduce the correlation between hand and cursor position. Finally, we replicate key results in human intracortical BMI users to show that cursor positional effects are also minimal in these participants.

## Results

### Subtracting expected cursor positional effects only helps when arm movements accompany BMI use

**Figure 1:**
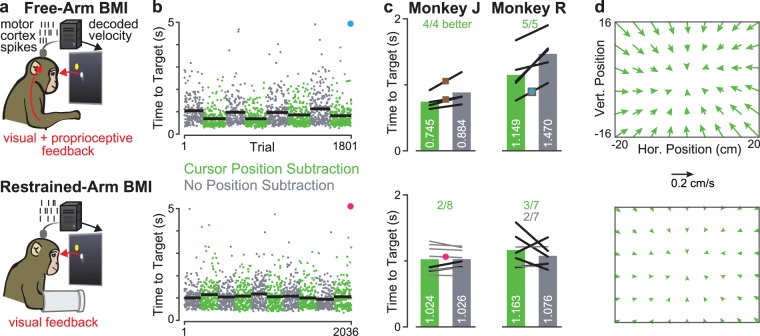
Cursor Position Subtraction only improves free-arm BMI performance. (**a**) Monkeys performed a Radial 8 Target Task using a BMI cursor controlled via decoded motor cortical spiking activity. Closed-loop performance was tested in two behavioral contexts: with the contralateral-to-arrays arm unseen but free to move (top row of all panels), or with this arm restrained (bottom row). (**b**) In each context, we compared variants of the velocity Kalman filter with and without subtracting the expected neural activity due to the cursor’s position. Example performance data is shown for one free-arm and one restrained-arm experiment session (datasets R.2013.01.28 and J.2013.11.15). Each point shows one successful trial’s time to target; its color denotes whether it was during a block with (green) or without (grey) Cursor Position Subtraction. Horizontal black bars show mean time to target for each block. (**c**) Aggregate performance across multiple experiment sessions. Each line compares the mean performance for a given session with (left) and without (right) Cursor Position Subtraction. Bolded lines denote datasets where the performance was significantly different (p < 0.01, rank-sum test between the two decoders’ distributions of times to targets). Fractions count how many of the sessions’ comparisons each decoder type won (i.e., had significantly lower times to target). Bar plot heights show mean performance across datasets. Datasets in which Cursor Position Subtraction was fit directly from a hand-controlled block (rather than from a closed-loop BMI recalibration block) are marked with brown squares. The example datasets from panel b are shown with corresponding colored circles. (**d**) The effects of Cursor Position Subtraction on the decoders from the two example datasets in panel b are shown as vector fields which indicate, for a given workspace location, what velocity would be added to the decoded velocity if the cursor was located there. These added dynamics pushed roughly towards the workspace center and were much stronger in the free-arm context.

Cursor Position Subtraction improved decoder performance when monkeys were free to move their arm during BMI use, but this operation did not improve performance in the absence of overt arm movements. Specifically, we performed head-to-head comparisons of velocity Kalman filters both with and without Cursor Position Subtraction in two contexts: with the monkey’s arm free to move, and with their arm restrained (Fig. 1a). Importantly, we as the experimenters did not require the monkeys to move their arm during BMI use, but they almost always did so when their arm was not restrained. This is unsurprising given that this was the behavior used to initially seed the free-arm BMI decoder. Within each context, we fit decoders with and without Cursor Position Subtraction from the same training data and then tested them in alternating blocks. Figure 1b shows trial-by-trial performance on a Radial 8 Target Task from example free-arm and restrained-arm experiment sessions, while Fig. 1c shows summary metrics for all sessions in two monkeys.

When the animals were free to move their arm during BMI use, times to target were significantly lower when Cursor Position Subtraction was used. This effect was robust in all four monkey J experiment sessions (‘datasets’; 4,563 total trials) and all five monkey R datasets (5,649 trials). This was the case both for decoders trained directly from arm reaching data (three datasets) and decoders recalibrated from closed-loop BMI data collected while the monkey performed the task with an initial arm reach-trained BMI decoder (six datasets). When comparing all datasets, Cursor Position Subtraction reduced grand mean of means time to target by 15.7% in J and 21.8% in R. This online performance benefit due to Cursor Position Subtraction is consistent with the results from Supplementary Figures 3 and 4 of^19^, which were also obtained in a free-arm context.

However, when the monkeys were not allowed to move their arm during BMI use, the outcome of the decoder comparisons was markedly different. Only two of eight J datasets (10,095 total trials) and three of seven R datasets (8,708 trials) showed a significant improvement when using Cursor Position Subtraction, and two of the seven R datasets showed a significant *reduction* in performance when using Cursor Position Subtraction. The grand mean time to target was 7.5% slower in monkey R when performing Cursor Position Subtraction, and the two decoding methods performed essentially the same for monkey J. To rule out performance ceiling effects in monkey J, we deliberately decreased overall decoder quality in several datasets (for both decoders with and without Cursor Position Subtraction) by using suboptimal decoder calibration methods and excluding electrodes from the decoders (see Methods). This did not change the outcome: the two individual datasets where Cursor Position Subtraction slightly improved performance were among those in the top half of performance and did not have any electrodes dropped.

To try to better understand why Cursor Position Subtraction did not appear to improve performance in the absence of overt arm movements, we first visualized what influence this operation exerted on the decoded velocity. Figure 1d shows representative vector field examples showing the effect of Cursor Position Subtraction on decoded velocity as a function of where in the workspace the cursor was. In the free-arm context, both monkeys’ Cursor Position Subtraction decoders added a modest velocity vector directed approximately towards the center of the workspace. These vector fields increased in magnitude near the workspace boundaries, and across different sessions’ decoders the maximum magnitude within the workspace ranged from 2.04 to 3.97 mm/s (3.06 ± 0.97, mean ± s.d.) for monkey J and 1.88 to 2.43 mm/s (mean 2.15 ± 0.21) for monkey R. This result is consistent with the attractor points described in^25^ for position-velocity Kalman filters initially seeded from arm movements (but then updated during BMI use without overt arm movements).

In contrast, the cursor position’s effect on velocity was very small in the restrained-arm context: the maximum magnitude ranged from 0.65 to 1.34 mm/s (0.94 mean ± 0.28 s.d.) for J and 0.30 to 0.76 mm/s (mean 0.53 ± 0.18) for monkey J. These results show that in the absence of arm movement, a linear mapping from cursor position to firing rates explained little variance in the training data. Consequently, the Cursor Position Subtraction operation that was fit from these data had minimal effect on decoded velocity. This in turn explains why we observed little difference between the standard and Cursor Position Subtraction restrained-arm decoders.

### Cursor position-related modulation is stronger during hand-controlled than restrained-arm BMI use

The previously described differences in the Cursor Position Subtraction velocity vector fields between free-arm and restrained-arm decoders imply weaker position-correlated neural modulation in the restrained-arm context. However, this interpretation has several limitations or other possible explanations: positional effects could be small within the limited range of positions sampled during the Radial 8 Target Task; position-related modulation could be outside the two neural dimensions that affect the decoder^26,27^; and/or position-related neural modulation could be poorly described by a linear fit. To more thoroughly investigate cursor positional effects without these limitations, we performed a set of experiments where monkeys performed a Random Target Task in which longer target hold epochs occurred across a larger span of the workspace. To directly compare positional effects during arm movements versus restrained-arm BMI use, during each experiment session the monkey first performed the task using a restrained-arm BMI, and then with his hand.

We grouped trials based on the workspace location of that trial’s target and examined firing rates (trial-averaged during the target hold epoch) within each of these workspace ‘tiles’. To quantify the upper bound of how much hold period firing rates varied as a function of cursor position without assuming a particular relationship (e.g., linear tuning or effect on the decoder), we measured each electrode’s firing rate difference between holding the cursor in the highest-rate tile and the lowest-rate tile. To provide context for these magnitudes, we also report them as a percentage of the electrode’s firing rate range during movements, calculated across both hand-controlled and restrained-arm task performance.

**Figure 2:**
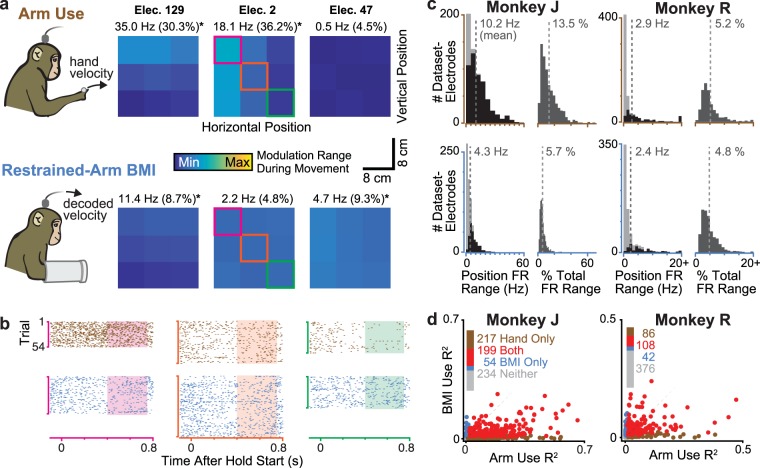
Effector position affects firing rates more during arm use than restrained-arm BMI use. (**a**) Motor cortical correlates of reaching hand position (top row) and BMI cursor position (bottom row) during Random Target Task target hold epochs for three example electrodes (columns). For this analysis, the workspace was divided into nine equally-sized tiles and trials were grouped based on target location. Each tile is colored based on the electrode’s mean firing rate during the end of the corresponding trials’ target hold periods. Each electrode’s colormap is normalized to its firing rate range across BMI and arm movement conditions and is fixed across the arm and BMI plots. Differences between the highest and lowest rate tiles in Hz (and as a percentage of the electrode’s firing rate range) are written above each plot; asterisks denote significant differences (p < 0.001, shuffle test). Dataset J.2013.04.24. (**b**) Example spike rasters during arm (brown) and BMI (blue) use. Trials are grouped by target location tile, with axis colors matched to the corresponding electrode’s tiles in panel a. Shading shows the epoch analyzed in the other panels. (**c**) Histograms of electrodes’ positional effects during arm (top row) and BMI use (bottom row), quantified as firing rate differences between highest and lowest-rate tiles (left) and as a percentage of firing rate range during movements (right). Each electrode contributes one point per dataset. Data aggregated across all four of each monkey’s datasets. For the rate difference histograms only, within-bin shading shows the number of dataset-electrodes with (dark) or without (light) significant differences. Note the different axis limits for each monkey. (**d**) Each point corresponds to one dataset-electrode whose firing rate modulated in response to movement during at least one of the behavioral contexts. Each dataset-electrode’s coordinates show how much of its firing rate variance was explained by hand position (linear regression) during arm use (horizontal) and cursor position during BMI use (vertical). Points’ colors denote whether a significant fraction (p < 0.001, shuffle test) of variance was explained during arm use only (brown), BMI use only (blue), both contexts (red), or in neither context (gray). Counts of how many points belong to each category are reported next to the legend.

Figure 2a shows firing rate as a function of workspace position for three example electrodes during one dataset (‘dataset-electrodes’) in both behavioral contexts, with single-trial spike rasters shown in Fig. 2b. These examples were chosen to illustrate position-correlated modulation in: both contexts (elec. 129, but note the greater modulation during arm use); during hand control only (elec. 2); and during restrained-arm BMI only (elec. 47). Figure 2c shows histograms summarizing these measurements across the four datasets collected in each monkey (J: 2,681 arm trials total, 3,796 BMI trials; R: 4,442 arm trials, 3,430 BMI trials). During arm use, 516/768 (67.2%) of monkey J’s dataset-electrodes showed a significant firing rate difference between the highest and lowest rate tiles (p < 0.001, rank-sum test). The mean of these differences across dataset-electrodes was 10.2 Hz (13.5% of movement-epoch range). During restrained-arm BMI use, hold-epoch firing rates varied substantially less across workspace position tiles: 279 (36.3%) showed significant modulation, with a mean difference of 4.3 Hz (5.7%).

Monkey R’s results also showed smaller positional effects during restrained-arm BMI use, although in this monkey the differences between contexts were smaller: during arm use 253/695 (35.0%) of electrodes showed a significant positional effect, with a mean difference of 2.9 Hz (5.2%). In the restrained-arm BMI context, 150 (21.6%) of dataset-electrodes had significant differences, with a mean difference of 2.4 Hz (4.8%). We note that monkey R had fewer electrodes recording good spike signals; this worse neural signal quality likely contributed to his smaller number of electrodes exhibiting a positional effect, and its smaller magnitude. These results extend those of the previous section by showing that across a wider variety of workspace positions, positional effects are stronger during arm use than restrained-arm BMI use. This further supports the hypothesis that consequential cursor positional effects only emerge when arm movements accompany BMI use.

Most electrodes with strong positional tuning showed relatively linear relationships between workspace position and firing rate, like in the Fig. 2a examples. We therefore quantified the single-trial firing rate variance explained by a linear fit to the cursor’s (horizontal, vertical) position. While simplistic, this analysis allows us to directly compare these results to how the position feedback subtraction operation of decoders like ReFIT would model the cursor positional effect, and to the descriptions of such behavioral correlates often reported in neurophysiology studies (e.g.,^9^). Whereas in the above tile analysis we included all available electrodes, here we attempted to restrict the analysis to the subset of electrodes with meaningful task-related modulation, which we defined as a significant firing rate change during movement initiation. The reasoning for this difference is that in the former analysis, we sought to describe what position-correlated activity is detected across the arrays; this is what a BMI system will have to work with. In this latter analysis we are categorizing how electrodes’ responses relate to arm reaching versus BMI cursor movements, and thus are less interested in electrodes that don’t modulate during either of the tasks.

Figure 2d shows that substantially more hold-epoch firing rate variance was explained by cursor position during arm use (which is equivalent to the hand’s position in the vertical plane) than by cursor position during restrained-arm BMI use. Amongst the 416 monkey J dataset-electrodes with significant variance explained due to cursor position during arm use (including those tuned during both contexts), the coefficient of determination ranged from 0.012 to 0.659 (mean 0.152). During the restrained-arm BMI context, only 253 dataset-electrodes had significant variance explained by cursor position, with a R^2^ range of 0.011 to 0.264 (mean 0.0493). For monkey R, the corresponding statistics were: 194 dataset-electrodes with significant R^2^ during arm use, ranging from 0.008 to 0.472 (mean 0.0758); 150 dataset-electrodes with significant R^2^ during BMI use, ranging from 0.010 to 0.240 (mean 0.0526). Of the dataset-electrodes with significant variance explained by cursor position in at least one context, only a minority (11.5% in J and 17.8% in R) had variance explained only during BMI use; the rest were roughly equally divided between having significant variance explained only in the hand-controlled context or in both contexts. These results are consistent with the prior tile analysis and demonstrate that there are stronger motor cortical correlations with the position of the hand during arm use than with the position of a BMI cursor in the absence of arm movements.

### Movement-epoch position-related modulation differences

In the previous section, we focused on positional effects during the target hold period. This minimizes the confounding effects of movement generation: movement-epoch activity includes efferent command components that could be different in different parts of the workspace, whereas active hold-period activity may better isolate possible neural correlates of the subject’s awareness of the effector position (along with any motor cortical efferents related to maintaining the hold posture during arm use). A more engineering-minded reason for examining hold epoch data is these are used to fit the Cursor Position Subtraction model of the ReFIT decoder, and one goal of this study was to verify whether this decoder operation was useful in the absence of overt arm movements. Nonetheless, we wanted to also compare movement-epoch positional effects between arm and BMI use, especially in light of evidence that the motor system operates in different regimes between moving and holding-in-place during both arm^11,28–30^ and BMI use^27^.

**Figure 3:**
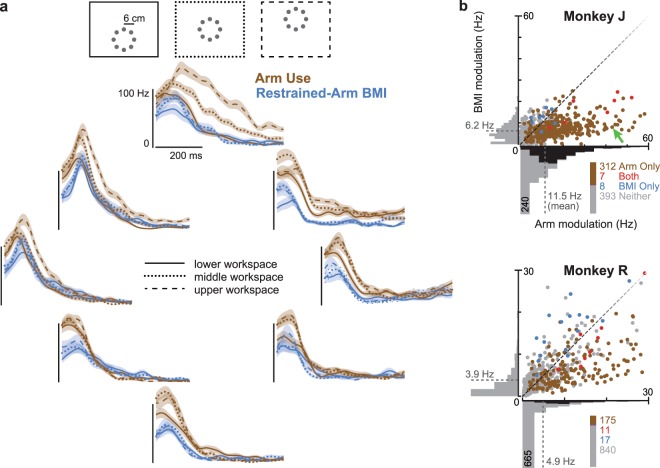
Peri-movement position-related modulation is greater during arm than BMI use. (**a**) Monkeys made Radial 3 × 8 Target Task out and back movements in each of three workspaces; a vertical task axis target arrangement is illustrated at the top. Firing rates are shown for an example electrode (J.2013.04.22, elec. 116) when moving either the arm (brown) or BMI without accompanying arm movement (blue). Each plot corresponds to outward movements towards a target in the direction corresponding to the plot location. Different line styles denote movements to different target sets centered on one of three locations within the overall workspace. Trial-averaged firing rates are shown from 40 to 600 ms after target onset, and shading shows s.e. This dataset-electrode’s maximal ‘workspace modulation’ was 42.9 Hz during arm movements towards the upper target, and 8.0 Hz during BMI movements towards the upper-right target. (**b**) Quantification of workspace-related firing rate modulation across all dataset-electrodes for each monkey (4 monkey J datasets, 6 R datasets). Each scatter plot point corresponds to one dataset-electrode. Its coordinates show the maximal workspace modulation during arm movement (horizontal position) and during BMI movement (vertical position). Points’ colors denote whether there was significant (p < 0.001, shuffle test) workspace-related modulation during arm use only (brown), BMI use only (blue), both contexts (red), or in neither context (gray). Counts of how many points belong to each category are reported next to the legend. The dataset-electrode from panel a is marked with a green arrow. Marginal histograms are each shown for behavioral context. Within-bin shading denotes the number of dataset-electrodes with (dark) or without (light) significant workplace modulation. Distribution means are marked by the dashed lines.

We examined movement-epoch position-related differences in a different set of experiments in which the monkeys performed a ‘Radial 3 × 8 Target Task’ using either their hand or a restrained-arm BMI. This task consisted of alternating blocks of Radial 8 Target sets such that the radial and center targets of each set were located in different areas of the workspace (Fig. 3a). The task design allowed us to compare neural activity between movements with the same extrinsic direction and distance, but in different parts of the workspace. Importantly, despite having the same vector from movement origin to target, arm movements in different workspaces of this task are biomechanically distinct; this is one reason why we would predict considerable position-related differences. Data were aggregated across four monkey J datasets (2,377 total arm trials and 2,314 BMI trials) and six monkey R datasets (2,998 arm trials and 2,618 BMI trials).

We quantified how neural activity differed between movements in the same direction but within different parts of the workspace. Specifically, for each of the eight outward target directions, we measured the firing rate from 0 to 600 ms after target onset, averaged separately for trials from each of the three target sets (i.e., in the three different workspace areas). We then defined a ‘workspace modulation’ metric as the difference magnitude between the target sets with the highest and lowest rates (e.g., the maximum minus the minimum firing rate amongst leftward reaches in the lower, middle, or upper workspace). Figure 3b shows each dataset-electrode’s maximal workspace modulation across the eight movement directions during either arm use or restrained-arm BMI use. These movement-epoch results were consistent with the previously described differences in hold-epoch positional effects. During arm movements, 319/720 (44.3%) of monkey J’s dataset-electrodes and 186/1043 (17.8%) of monkey R’s dataset-electrodes showed significant modulation differences across movements in the same direction but in different areas of the workspace (p < 0.001, shuffle test). In contrast, during restrained-arm BMI movements only 15 (2.1%) of monkey J’s and 28 (2.7%) of monkey R’s dataset-electrodes exhibited significant workspace modulation. Thus, positional effects were much weaker during BMI use than arm use, both when moving towards a target and when holding the cursor over the target.

### Firing rates correlate with both cursor and hand positions during free-arm BMI use

Thus far, we showed that hand position had stronger motor cortical correlates than restrained-arm BMI cursor position. We next asked, does the cursor position exert any additional influence on firing rates when monkeys are allowed to move their arm during BMI use? The answer to this question could be relevant to future patients using movement neural prostheses who still have some residual motor function. However, for the purpose of answering this question, a “problem” was that the hand and cursor positions were strongly correlated when the monkeys used high performing BMI decoders. This was because, as previously described, the monkeys had adopted a strategy of making actual arm movements when controlling the cursor in a free-arm BMI context.

To address this problem, we intentionally trained lower performing decoders for the next set of experiments by using only a subset of electrodes and forgoing the beneficial Cursor Goal intention estimation training data correction^19^. Our motivation for deliberately degrading the decoder was that this would reduce the correlations between hand and cursor movements (i.e., the decoder would not as accurately infer the monkey’s intended movement, causing the hand and cursor to diverge over the course of the movement). Decorrelating the hand and cursor positions would allow us to better tease apart their separate neural contributions. Although only a subset of electrodes’ activity causally moved the BMI cursor, all of the electrodes were included in the following neural analyses.

**Figure 4:**
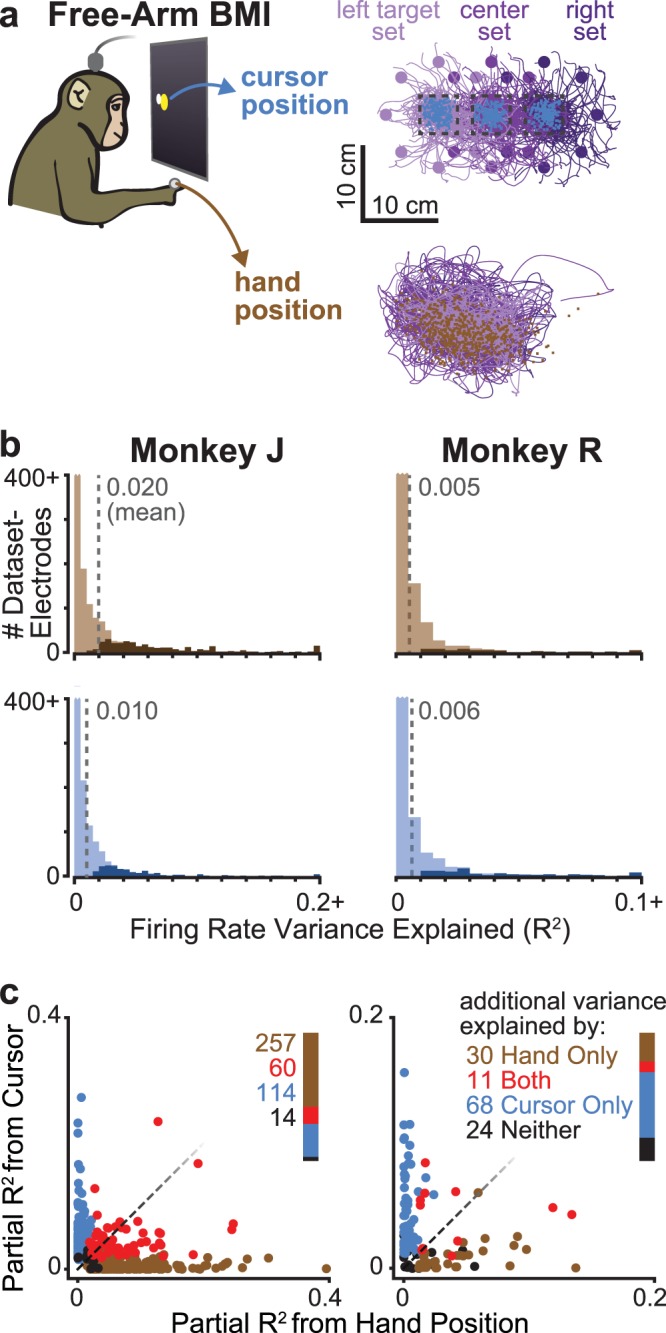
Hand and cursor positions both affect neural activity during free-arm BMI use. (**a**) We examined the separate influence of cursor and hand positions on firing rates during the free-arm BMI use context, during which they are somewhat decoupled. Monkeys performed a Radial 3 × 8 Target Task. Simultaneous cursor (top) and hand (bottom) trajectories are shown for example dataset R.2013.11.19, color-coded based on which of the three target sets they belong to. Firing rates were compared during hold periods when the cursor was within a center target’s acquisition area (dashed boxes). Every trial’s cursor and hand positions during the analysis epoch are shown with blue and brown points, respectively. For clarity, only a subset of trials’ trajectories is shown. (**b**) Histograms showing what fraction of each electrode’s firing rate variance was explained by 1-D cursor position (blue) or hand position (brown) along the workspace dimension that the center target locations varied over (e.g., horizontal position in panel a). The number of dataset-electrodes with significant variance explained within each bin are denoted with an opaque shade of the color. Data are aggregated across all eight monkey J and seven R datasets. For visual clarity, the leftmost bins are cut off at the top. (**c**) Each point represents a dataset-electrode for which a significant fraction of neural variance was explained by cursor or hand position (or both). We computed the partial correlation between the cursor position and firing rate, accounting for shared variance with hand position (vertical coordinate) and, conversely, the partial correlation between hand position and firing rate, accounting for the cursor position (horizontal coordinate). Colors denote whether additional neural variance was explained by hand position only (brown), cursor position only (blue), both cursor and hand positions (red), or neither (black). Counts of how many dataset-electrodes belong to each category (i.e., have partial correlations with that parameter beyond the variance explained by the correlated movement of the cursor and hand) are reported next to the legend.

In these free-arm BMI experiments, the monkeys again performed the Radial 3 × 8 Target Task. We restricted our analyses to only the target hold periods of trials to the central target of each of the three target sets (Fig. 4a). This experiment design was chosen for two reasons: first, when monkeys used a BMI to control the cursor in the wider workspace of the Random Target Task, there were more times when their hand was out of sight of the hand tracking system; analyzing the center hold of Radial 3 × 8 Target Task data reduced the prevalence of such drop-outs. Second, this design addresses a potential concern with the previous tasks’ hold-epoch analyses: it could be that some of the position-correlated hold epoch activity observed was due to preparing the subsequent movement. This is because the subsequent target after acquiring radial targets in the standard Radial 8 Target Task is always the center target. In the Random Target Task, the next target is not known, but it is more likely to be towards the workspace center. Examining Radial 3 × 8 Target Task center target hold activity eliminates this concern, because the possible next targets are symmetrically spaced in all directions. We do note, however, that even if preparing-while-holding contributes to the positional effects described earlier, this would not explain why the positional effects were stronger during hand control compared to restrained-arm BMI control (ref.^31^ showed that preparatory activity is similar between these two contexts in these same two monkeys).

Both monkeys were able to perform the free-arm BMI Radial 3 × 8 Target Task despite the intentionally degraded decoders. Across his eight datasets, monkey J successfully completed between 96.3% and 100% of trials (mean of 97.9%), with a mean time to target of 858 ms (grand mean of individual datasets’ means). Monkey R’s success percentage ranged from 84.5% to 99.3% (mean 94.0%) with a grand mean time to target of 1,427 ms. We analyzed center target hold periods from a total of 3,912 monkey J trials and 4,399 monkey R trials. Since cursor position essentially only varied along one dimension in these data (the task axis connecting the three Radial 8 target set centers), we examined the relationship between hold epoch firing rates and both cursor and hand positions along this axis. These two variables were quite decoupled: across monkey J datasets, the within-session correlation between hold-epoch cursor and hand positions ranged from −0.128 to +0.162 (mean −0.017). Across R datasets, the correlation ranged from −0.560 to +0.873 (mean −0.04).

Both of these kinematic variables were correlated with firing rates: Fig. 4b shows histograms of firing rate variance explained using just BMI cursor position or hand position: 327/1536 (21%) monkey J and 63/1344 (5%) monkey R dataset-electrodes’ activity significantly varied with hand position (p < 0.001, shuffle test), while 176 (11%) J and 97 (7%) R dataset-electrodes’ activity significantly varied with BMI cursor position. Neural variance explained in the Radial 3 × 8 Target Task was smaller than in the Random Target Task: among those electrodes with significant modulation to hand or cursor position, monkey J’s mean R^2^ was 0.071 for hand and 0.050 for cursor, and monkey R’s was 0.042 for hand and 0.043 for cursor. This was expected given that the 1D cursor and hand positions’ trial-to-trial variability in the Radial 3 × 8 Target Task was much smaller than the 2D position variability in the Random Target Task.

The observation that there were significant and distinguishable neural correlations with both hand and cursor positions during free-arm BMI use allowed us to turn to the more interesting question that motivated this last set of monkey experiments: did both cursor and hand positions contribute to firing rates, beyond their shared covariation? This analysis was restricted to the 445 monkey J dataset-electrodes and 133 R dataset-electrodes with significant variance explained by either cursor or hand positions in the previous analysis. Figure 4c shows these dataset-electrodes’ partial neural variance explained by hand position (beyond that explained by its covariation with cursor position) on the horizontal axis, and partial variance explained by cursor position (beyond that explained by its covariation with hand position) on the vertical axis. The key take-away from this analysis is that both the hand and cursor positions did separately matter to the ensemble population activity.

The fact that hand position explained less additional variance than cursor position in monkey R should not be interpreted as inconsistent with the previous results, and does not imply that the cursor was “more important” from the perspective of neural modulation. This is because this particular experiment was designed to answer whether or not both the hand and cursor influenced neural activity during free-arm BMI use, rather than to quantitatively compare which had more influence. The reason for this limitation is that the task and analysis are not “balanced”, by which we mean that 1) there was more BMI cursor position variability than hand variability, and 2) some of the hand variability was in the dimension orthogonal to the task axis and thus not included in the partial correlations, whereas almost all of the cursor position variance was by definition along the task axis since the cursor is what was used to perform the task. Both of these phenomena can be seen in the example dataset kinematics shown in Fig. 4a. To summarize: the previous experiments showed that hand position during arm use has stronger neural correlates than cursor position during BMI use; this experiment further shows that both hand and cursor positions do separately influence firing rates when they are decoupled during BMI use that is accompanied by arm movements.

### Cursor positional effects are also minimal in humans with paralysis

**Figure 5:**
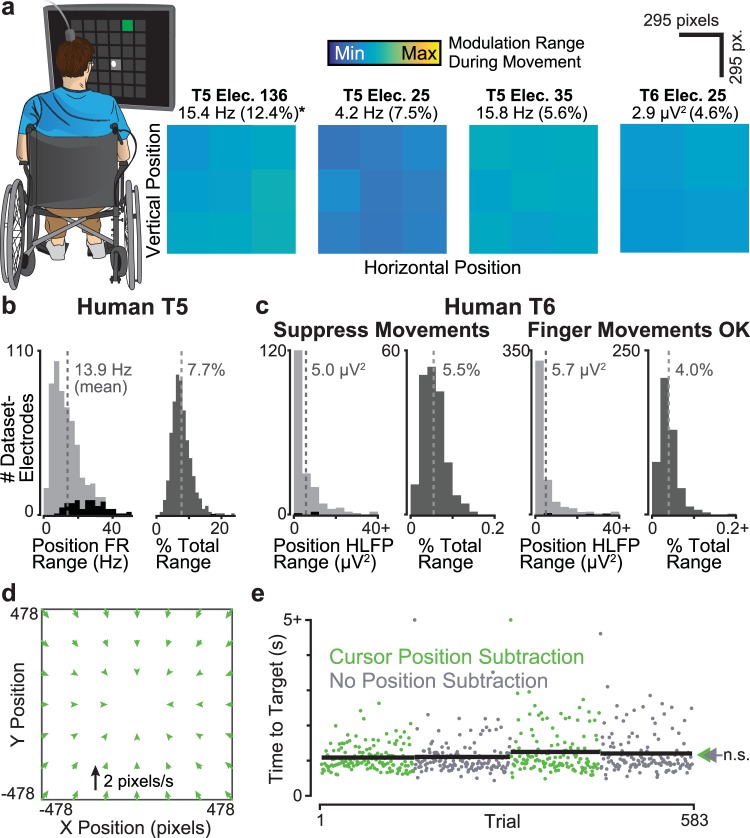
BMI cursor position correlates in human motor cortex are small. (**a**) An analysis similar to that of Fig. 2 was performed using motor cortical data from two human clinical trial participants performing a Grid Task with a BMI cursor. Trials were grouped by dividing the target locations within the workspace into nine (participant T5) or four (participant T6) tiles. The plot shows, for four example electrodes, trial-averaged neural activity immediately prior to target selection. Data are from datasets T5.2016.10.13 and T6.2014.07.02. Note that for T6, the principal BMI control signal was high frequency LFP power (HLFP), rather than spike rate. (**b**) Data from ‘T5’, who has spinal cord injury and is unable to volitionally move his arms, presented as in Fig. 2c. (**c**) Similar analysis for ‘T6’, who has ALS and was still able to volitionally move her hands. We collected data in two different behavioral contexts: in the movement suppressed context (left), we asked T6 to avoid moving her hand. In the regular context (right), she was free to (and did) make finger movements during BMI use. (**d**) We performed a closed-loop decoder comparison with and without Cursor Position Subtraction (dataset T5.2017.06.28). This panel shows the effect of Cursor Position Subtraction on cursor velocity as in Fig. 1d. (**e**) Trial-by-trial closed-loop Radial 8 Target Task performance, shown as in Fig. 1b. Two points with values of >5 s are drawn at the plot ceiling. Arrows show each decoder’s mean time to target.

We believe that macaques are an excellent animal model for investigations that are intended to ultimately be applied to BMI systems for human use. Here, however, we were in the advantageous position of being able to further bolster our findings with human BMI data. Specifically, we analyzed previously-collected data (recently reported in^2^) where two clinical trial participants performed a typing-like Grid Task in which they used a BMI that decoded attempted arm or finger movements to acquire a cued target amongst a grid of possible targets (Fig. 5a). Although the human Grid Task has minor differences compared to the monkey Random Target Task, it is essentially similar: the participant had to move the cursor to randomly appearing targets (discretely) spanning a workspace and then select the cued target. An important difference is that the people used a discrete ‘click’ command to select the target, whereas the monkeys merely held the cursor over the target. We examined how human neural activity preceding the click selection varied as a function of where in the workspace the target was (Fig. 5a), similarly to the earlier analysis of hold-period monkey cursor positional effects shown in Fig. 2a–c. There were several noteworthy differences between participants, so we will describe each of their results in turn.

Participant ‘T5’ had tetraplegia due to spinal cord injury and lacked volitional control and sensation of his arms. Therefore, even though he used arm movement and hand squeeze imagery to control the cursor velocity and click, his data should be unaffected by arm somatosensory and proprioceptive feedback effects. T5 had two 96-electrode arrays chronically placed in motor cortex, and most of these electrodes were able to record action potentials. Both the online BMI velocity decoding and the subsequent analyses reported here were therefore performed on threshold crossing firing rates, like in the previous monkey results. Figure 5b quantifies cursor positional effects in T5’s neural activity. Neural modulation was modest: across the 3 datasets (1474 trials), the mean firing rate difference between the tiles with the highest and lowest activity was 13.9 Hz. This firing rate difference was significant for 83/576 (14.4%) of dataset-electrodes (p < 0.001, rank-sum test). When firing rate changes were normalized to each electrode’s range, this maximum positional effect corresponded to a mean of 7.7% of overall task-related firing rate changes.

Participant T6’s data differed in two substantial ways. First, she had a single 96-electrode array placed in motor cortex, which had worse signal quality resulting in few high-amplitude action potentials. To overcome this and extract more useful information about her movement intentions, an additional neural feature was derived from the raw voltage measurements: high frequency local field potential power (HLFP). This HLFP was the dominant signal used for T6’s velocity decoding, and it enabled high BMI cursor control performance^2^. The present analyses therefore examined this HLFP feature rather than threshold crossings. Second, T6, who had ALS, was still able to move her hands at the time of these experiments. She used imagery of finger movements contralateral to the array to command cursor velocity and imagined squeezing the hand ipsilateral to the array to click. To assess whether T6’s ability to make overt movements affected her BMI performance, Grid Task data was collected under two conditions: with T6 asked to suppress her hand and finger movements, or with T6 free to move her hands and squeeze her fist (which she did).

Figure 5c shows T6’s cursor positional neural effects, which were similar between the movement-suppressed and free-to-move conditions. We speculate that this could be because finger movements elicited less motor cortical feedback than the monkeys’ larger arm movements. When suppressing movements (two datasets, 224 trials), the mean difference between the highest and lowest tiles was 5.7 μV^2^, which corresponded to a mean of 5.5% of electrodes’ overall task-related activity range. When T6 was free to move during the BMI task (five datasets, 944 trials), the mean positional activity difference was 5.0 μV^2^, or 4.0% of the task-related range. This position-related modulation was significant in 3/192 (1.6%) of dataset-electrodes during the movement-suppressed condition and 5/480 (1.0%) of dataset-electrodes during the free-to-move condition. The weak cursor positional effects observed in T6’s data are similar to those of T5, with the caveat that two different motor cortical signals (high-frequency LFP power versus threshold crossing spike rate) were analyzed in these two participants. The results of these offline analyses of human BMI user data were consistent with the monkey restrained-arm results, both in the case of BMI use without overt movement and, in the case of T6, with some finger movements. This strongly predicts that the effect of BMI cursor position on decoding intended movements in people with paralysis would be inconsequential.

Finally, to further increase our confidence in this prediction, we performed a closed-loop decoder comparison during one research session with participant T5. This comparison was modeled on the earlier monkey decoder comparisons. First, an initial decoder was trained using the ReFIT protocol (open-loop calibration followed by closed-loop calibration). The data collected from the closed-loop block was then used to fit two final decoders from this same training data: one with and one without Cursor Position Subtraction. Figure 5d plots a vector field showing the effect of cursor position on velocity for the decoder with Cursor Position Subtraction. Consistent with the small positional effects on neural activity described in the previous offline analyses, the magnitude of positional effects on decoder velocity were also minimal: the maximum magnitude within the workspace was only 0.98 pixels/second. T5 used these two decoders to perform a Radial 8 Target Task in an alternating ABAB block design (Fig. 5e). As predicted, there was not a significant difference in online BMI performance: times to target were 1561 ± 572 ms (mean ± s.d.) with the Cursor Position Subtraction decoder operation and 1545 ± 578 ms without it (p = 0.43, rank-sum test).

## Discussion

We have presented multiple complementary lines of evidence that support the same conclusion: motor cortical correlates of the position of a two-dimensional BMI-controlled cursor, *without accompanying arm movements*, are weak and unlikely to be a meaningful nuisance variable during decoding. We first showed that subtracting the expected cursor position’s neural contribution as described in^19^ did improve BMI performance if the monkeys were allowed to move their arm during BMI use, but not when the BMI was used in the absence of arm movements (Fig. 1). This suggested that the strong cursor positional effects in the free-arm context were due to proprioceptive and/or efferent activity related to the arm being in different parts of the workspace.

Further monkey experiments comparing cursor positional effects when the cursor was controlled either using the hand or with an arm-restrained BMI (Figs 2, 3) confirmed that neural modulation was stronger during arm use. However, we observed that some electrodes’ activity did covary with cursor position even in the absence of arm movements. This suggested that both BMI cursor and hand positions could influence motor cortical activity if both hand and cursor moved during BMI use, and we indeed observed this during free-arm BMI experiments (Fig. 4). Together, these pre-clinical animal model results suggested that for clinical BMI cursor control applications such as typing and using a computer^32,33^, cursor position will minimally impact neural signals. We then presented further evidence which supports this prediction by analyzing two human participants’ previously-collected BMI cursor task data, as well as performing a new closed-loop decoder comparison in one participant (Fig. 5).

### Implications for BMI decoder design

We set out to test the assumption that the sensorimotor system’s awareness of the BMI cursor position (via vision and/or an internal model) would have motor cortical correlates that decoders should account for. However, our results indicate that for BMIs that are not accompanied by overt movement, these effects are minimal and will not interfere with a decoder’s ability to infer movement intentions. Zhang and Chase^18^ recently speculated that ReFIT^19^ performs so well because its position decoding implementation allows the decoder to still work as a second-order physical system. While this may very well be an advantage of a physics-obeying position-velocity Kalman filter compared to a position-velocity Kalman filter that cannot be expressed as a simple physical system, our results suggest that the main advantage of ReFIT over a velocity-only Kalman filter (with similar training data improvements) is that it accounts for a nuisance term that is present when the BMI user’s arm is allowed to move.

Arm movements accompanying BMI use are not expected in nearer-term clinical BMI systems^1^ providing cursor control to people with paralysis or amputation. Nuisance variable mitigations such as Cursor Position Subtraction are therefore unnecessary in these applications, and we recommend against their use because such decoder operations introduce additional free parameters that are subject to neural non-stationarities or estimation errors, as noted in^24,25^. Consistent with this recommendation, the Cursor Position Subtraction operation was not used in our recent study in which the ReFIT decoder enabled people with paralysis to type using a virtual keyboard^2^. These results add to an emerging story that the effects of some forms of visual feedback on motor cortical activity may be less problematic for BMI decoding than originally predicted^27^ (but see^34^, which reports that seeing a BMI arm approaching a graspable object strongly modulates activity).

Our observation that a centering velocity vector (due to Cursor Position Subtraction) improved performance during free-arm BMI use raises the possibility that such effector dynamics may be beneficial for BMI control in at least some tasks. We have previously used an ad-hoc variant of this to improve performance^35^. Such ‘centering dynamics’ could be systematically investigated and optimized in future work by directly adding various centering velocity vector fields during arm-restrained BMI use.

### Interpretation limitations

We believe that the stronger positional effects observed during arm use and during BMI use accompanied by arm movements can be attributed to a combination of motor cortical control of holding the arm in different positions (i.e., different output) and proprioceptive feedback (i.e., different input). These results are consistent with previous reports that motor cortex is strongly modulated by arm position^9–15^ and that proprioceptive feedback about movements has stronger influence on M1 activity than visual feedback^36^. Both of these processes (between which our study does not disambiguate) are absent during restrained-arm BMI use. However, we cannot rule out that these differences are due to some cognitive change between when the sensorimotor system is or is not engaged in moving the arm.

Although we showed here that visually monitored BMI effector positional effects are minimal and can be ignored during decoding, it is unknown whether more anthropomorphic BMI effectors such as robotic arms^34,37^ will induce stronger motor cortical modulation related to effector state (e.g., arm position or joints conformation). This might be expected because these prostheses more directly tap into the sensorimotor system’s native arm-monitoring machinery. We also anticipate future BMI use cases where there will be proprioceptive feedback from the effector. There are ongoing efforts to make bidirectional BMIs that write in proprioceptive and somatosensory information^38,39^. Alternatively, the person’s own limb can be controlled via robotic exoskeletons^20,40^ or functional electrical stimulation of the muscles^4,41^. These situations may be more akin to the free-arm BMI context in this study, in that treating the expected contribution of the effector’s position as a nuisance variable could improve decoding movement commands.

A remaining question is, what caused the cursor positional effects observed during BMI use without accompanying movements? Likewise, what was the source of the cursor-specific neural variance in the free-arm BMI experiments? One possibility is that these are indeed motor cortical correlates of the subject’s internal state estimation of the cursor’s position. However, these small cursor position-correlated neural activity changes could also reflect the position of the user’s eyes. In these experiments, the monkeys were head-fixed but were free to fixate wherever they chose. The human participants were able to move their eyes and head. In practice, BMI users in these kinds of tasks will look at either the cursor or the target, which are highly correlated over the course of the experiment. During the target hold period, these positions are effectively the same. Previous work has shown modest gaze-related activity in PMd during reaching tasks^42–46^, and accounting for this effect can improve BMI performance^47^. However, here we also saw similar cursor position-correlated modulation in M1, which is not believed to track gaze^48^. Nonetheless, in this study we cannot rule out gaze-related (and head-related, for the human results) modulation. Future studies with enforced fixation and blanking periods without visual feedback would be needed to more definitively attribute cursor positional effects to an internal model rather than gaze and afferent visual information. However, it is possible that the experimental manipulation of enforcing eye fixation will itself increase the magnitude of its neural correlates^43,44^. Regardless of this lingering scientific question, from a BMI design perspective, it is reassuring that even if there are gaze effects in motor cortex during BMI use, they are small.

## Methods

### Research animals and human participants

This study involved two adult male rhesus macaques: monkey J (15 kg and 11 years old at the start of these experiments) and monkey R (13 kg, 7 years old). All experiments and procedures were approved by the Stanford University Institutional Animal Care and Use Committee and were performed in accordance with relevant guidelines and regulations. This study also includes two human participants, ‘T5’ and ‘T6’, who gave informed consent and were enrolled in the BrainGate2 Neural Interface System clinical trial (ClinicalTrials.gov Identifier: NCT00912041, registered June 3, 2009). This pilot clinical trial was approved under an Investigational Device Exemption (IDE) by the US Food and Drug Administration. Permission was also granted by the Institutional Review Boards of Stanford University (protocol #20804) and Partners Healthcare/Massachusetts General Hospital (2011P001036). All research was performed in accordance with relevant guidelines/regulations.

T5 was male, 64 years old at the start of this work, and right-handed. He was diagnosed with C4 AIS-C spinal cord injury ten years prior to this study. He retained the ability to weakly flex his left elbow, as well as inconsistent slight residual movements of upper and lower extremities and occasional involuntary spastic flexion. He retained neck and head movements and was able to speak. T6 was female, 51 years old at the start of this work, and right-handed. She was diagnosed with Amyotrophic Lateral Sclerosis (ALS) with motor impairment (ALSFRS-R functional scale rating of 16). She retained dexterous movements of the fingers and wrist, as well as the ability to move her head and speak, at the time of this study.

Multiple experiment sessions were performed with each monkey and participant. We will refer to the data from one experiment session as a ‘dataset’.

### Neural recording

Neural data were recorded using essentially similar chronic multielectrode arrays and recording hardware (Cerebus systems for monkeys, NeuroPort systems for humans, both by Blackrock Microsystems, USA). The human neural recording system, along with the BMI and experiment control system, form the BrainGate2 Neural Interface System (CAUTION: Investigational Device. Limited by US Federal Law to Investigational use).

Monkeys J and R had one 96-electrode array (10 × 10 layout of length 1 mm electrodes with 400 μm pitch/spacing) neurosurgically placed in the dorsal aspect of premotor cortex (PMd) and one array placed in the gyral primary motor cortex (M1). When we performed the key analyses of the study separately for M1 and PMd electrodes, we observed similar results; we therefore report results pooled across these areas. In each monkey, both arrays were contralateral to the arm used for reaching in the free-arm BMI and hand control behavioral contexts. J’s data were collected 43 to 65 months after array placement. R’s data were collected 16 to 40 months after array placement. Participant T5 had two 96-electrode Blackrock arrays (1.5 mm electrode length) placed in the hand knob area of dominant motor cortex. T6 had a single 96-electrode Blackrock array (1 mm electrode length) placed in the same area. Figure 1 of^49^ shows the exact locations of the arrays for both participants. Participant T5’s Grid Task data (Fig. 5a,b) were collected 2 months after his arrays’ placement, and the Radial 8 Target Task data (Fig. 5d,e) were collected 10 months after arrays placement. T6’s Grid Task data were collected 19-20 months after array placement.

Voltage signals were analog filtered from 0.3 Hz to 7.5 kHz and digitized at 30 kHz. In the monkey experiments this signal was then digitally filtered from 250 Hz to 7500 Hz, and a threshold crossing spike was detected whenever the voltage dropped below −4.5 times the root mean square (RMS) voltage. As is standard for BMI studies, we did not spike-sort to assign waveforms to putative individual neurons. The analyzed spikes therefore reflect both single- and multiunit activity. In the human experiments, the raw voltage signal was processed by the real-time Simulink system, which first applied a common average reference to each electrode within an array. The signal was then split into a spike band (250 Hz asymmetric FIR high pass filter optimized to extract spike activity from Utah arrays^50^), and a high frequency local field potential (HLFP) band (power in the 150 Hz to 450 Hz band-pass filtered signal). A voltage threshold was applied to the spike band to obtain threshold crossing spikes. For participants T5 and T6’s Grid Task experiments, these voltage thresholds were −50 and −95 μV, respectively. For T5’s Radial 8 Target Task decoder comparisons, the threshold was set to −4.5 × RMS, like in the monkey experiments. The firing rate plots in Fig. 3a (peristimulus time histograms) were constructed by smoothing spike time series with a Gaussian kernel with 20 ms s.d. and averaging firing rates across trials of the same behavioral condition.

### Experiment setup

Monkeys were trained to sit in a primate chair and perform a 2D cursor task in which they controlled the velocity of a virtual cursor either with their hand or with a BMI. The task was displayed using a Wheatstone stereograph and appeared in the fronto-parallel plane. The BMI task was performed in two different behavioral contexts: ‘free-arm’, in which the monkey could move one arm, or ‘restrained-arm’, in which the arm was gently restrained. The other arm was restrained in all experiments. In the free-arm BMI and hand-controlled tasks, the monkey could not see his reaching arm because it was occluded by the display mirrors. The position of the monkey’s hand (specifically, of an infrared-reflective bead taped across two of the fingers) was tracked at 60 Hz using a Polaris tracking system (Northern Digital). Prior to this study, both monkeys had at least a year of extensive experience performing hand-controlled and BMI tasks in all of the contexts tested; their behavior was stable over the course of the experiments.

In the human tasks, the person sat in a chair and performed cursor control tasks presented on a computer monitor in front of them. Research sessions were conducted in the participant’s home using a semi-portable cart-mounted system. Participant T6’s BMI data were collected in two different behavioral contexts: she was either free to make finger movements, or was asked to suppress her movements. Movement suppression was verified by measuring finger movements with a dataglove (5DT), as reported in^2^.

The monkey and human experiment flow and BMI systems were based on the same underlying platform, and were similar; we describe where there were noteworthy differences below. Experiments were implemented using a custom Simulink xPC/Realtime platform (Mathworks, USA), operating at a 1 ms clock, which enabled 1 ms precision timing for all data. The task was displayed on 120 Hz monitors, using MSMS (University of Southern California, www.mddf.usc.edu) for the monkeys and Psychophysics Toolbox (www.psychtoolbox.org) for the humans.

### Radial 8 Target Task

This task, as well as the others described below, consists of moving a virtual cursor to acquire virtual targets in a 40 cm wide by 32 cm tall workspace. In the Radial 8 Target Task, the target location alternated between the workspace center and one of eight radial locations (pseudorandomly chosen within 8-target sequences) equally spaced around a circle centered on the workspace center. The radial target centers were located 12 cm from the workspace center during blocks used for decoder calibration, and 8 cm from the center during the decoder evaluation blocks. We define the acquisition to the radial target and the acquisition of the center target as two separate trials. In the monkey variant of the task, only one target was visible at any given time.

The target was acquired by holding the cursor within the target acquisition area for a contiguous 500 ms. Leaving the target area did not fail the trial, but it did reset this acquisition time counter. The target acquisition area was a 4 × 4 cm square (5 × 5 cm for monkey J free-arm context) surrounding the (smaller) visual circular depiction of the target, with the following exceptions: during the first-pass closed-loop BMI blocks where the initial decoder was used (which was typically worse than the final decoder), the target area was 6 × 6 cm or occasionally 7 × 7 cm; this helped prevent the monkeys from becoming frustrated. For all tasks, the next target was displayed 20 ms after a trail success or failure. The trial time limits for the closed-loop decoder comparisons (Fig. 1) were 5 s. For other tasks and experiments, trial time limits varied depending on the monkey and dataset, but were always generous enough that time limit was not a limiting factor in the monkey’s performance; the occasional timed out trials (putatively due to disengagement from the task) were not analyzed.

One participant T5 Radial 8 Target Task dataset was specifically collected for this study to compare decoders with and without a ‘Cursor Position Subtraction’ operation. The workspace was 1920 pixels wide by 1080 pixels tall. The eight circular targets, each of radius of 50 pixels, were equally spaced around a circle of radius 409 pixels. All eight targets were always displayed, and the trial’s active target was shown with a different color (the other targets could not be selected). The active target was selected if the cursor center was held inside the target (the acquisition area was circular for the human variant of the Radial 8 Target Task) for a continuous 500 ms. The trial failed after 10 s.

For monkey decoder comparisons, each decoder was used in alternating blocks of approximately 200 trials per block (an ABABAB… design). T5’s decoder comparison consisted of a single ABAB block design session using a ReFIT decoder with (A) and without (B) Cursor Position Subtraction. To compare decoder performance, we used the metric of time to target, which is only calculated for successful trials. Overall success rates were 97.1% and 97.2% for monkeys J and R, respectively, and 99.8% for participant T5. For all three BMI users, task performance was sufficiently high that any failure was almost certainly attributable to task disengagement, rather than an inability to acquire the target. To exclude trials in which the animal or participant may have briefly disengaged from the task, we only analyzed successful trials that followed another successful trial. For the monkey datasets, we further avoided including data with dubious motivation at the very end of the session, right before the monkey chose to stop working. Specifically, we used the following block inclusion rule: if the animal did not finish the last AB ‘block-set’ of the experiment session, this block-set was excluded. If the animal did finish the block-set, then its last block was excluded.

### Radial 3 × 8 Target Task

A ‘Radial 3 × 8 Target Task’ variant was used for the Fig. 3 experiment in which monkeys performed the task using hand control and restrained-arm BMI control during the same experiment sessions, and in the Fig. 4 free-arm BMI experiments. During each of these experiment sessions, three different Radial 8 target sets were used; each of the target sets consisted of a center target and eight radial targets, but the entire target set could be shifted over in the workspace. Specifically, the center targets were located at either −7 cm, 0 cm, or +7 cm relative to the overall workspace center along a horizontal ‘task axis’, or −6 cm, 0 cm, or +6 cm along a vertical task axis. Radial targets were located 7 cm from the corresponding center target in the horizontal task axis arrangement and 6 cm from the center target in the vertical task axis arrangement. The smaller vertical task extent was to accommodate the animals’ reduced vertical range of arm motion. For this same reason, the topmost three targets of monkey J’s hand-controlled vertical axis Radial 3 × 8 Target Task were moved down by 1 cm to accommodate his slightly shorter arms.

The task axis was fixed for a particular dataset but was varied from day to day. For the free-arm BMI experiments, a total of seven datasets were collected with a horizontal task axis arrangement and eight datasets were collected with a vertical task axis arrangement. For the hand control + restrained-arm BMI experiments, there were five horizontal and five vertical task axis datasets. The three target sets were presented in approximately 100 trial blocks of the same target set, with the target sets interleaved throughout the session. Specific task details for these experiments are: the target acquisition area was 4 × 4 cm when the task was performed under hand control and 5 × 5 cm when performed under BMI control; the requisite target hold period was 800 ms; the trial time limit was 3 seconds during hand control and 5 seconds during BMI control.

### Random Target Task (monkeys)

At the start of each trial, the target appeared anywhere in a 24 × 24 cm region centered within the overall workspace. The target acquisition area was 4 × 4 cm when the cursor was hand-controlled and 5 × 5 cm when BMI-controlled. The monkey acquired the target by holding the cursor within this area for a contiguous 800 ms. The next target was presented 20 ms after the success or failure of a trial.

### Grid Task (humans)

We examined previously collected data from^2^ to measure cursor positional effects in human participants using a BMI to perform a Grid Task (Fig. 5a–c). A 1000 × 1000 pixel region centered within an overall 1078 × 1078 pixel workspace was divided into a 6 × 6 grid (T5 and T6) or 9 × 9 grid (T5 only) of equally-sized square gray targets. We analyzed T5 data from both grid sizes together since a different post-hoc division of trials by target regions was used for these analyses (‘tiles’, described later). Each target was selectable at all times, but for each trial, the correct target was prompted by being illuminated in green. A particular target could be selected either by holding the cursor over it for 1 s, or by commanding a ‘click’ (see “BMI decoders: human” methods below); both participants primarily used the click method. We only analyzed successful trials (98% for T5, 93% for T6). To reduce the effects of variability due to selection method, we only analyzed trials where the selection was made with a click (91% of successful trials for T5 and 87% for T6). Furthermore, since our analyses focused on neural differences based on where in the workspace the cursor was being held, we only analyzed trials where the cursor was held over the target for at least 150 ms prior to the click selection; this meant analyzing 58% (T5) and 94% (T6) of successful click trials.

### Dataset selection

In this section we describe how we chose which collected datasets to analyze. The decision to exclude a dataset was always made prior to performing any cursor positional effect neural analyses, and was instead based on disqualifying task behavior or if it was found that there was a technical problem with the data collection.

For the closed-loop monkey decoder comparisons (Fig. 1) we analyzed all collected datasets of the Radial 8 Target Task where the monkey completed at least two pairs of blocks with each decoder (i.e., at least “ABAB…”). Twelve monkey J datasets were analyzed (two were excluded due to insufficient trials). Twelve monkey R datasets were analyzed (two were excluded due to insufficient trials).

For the Random Target Task experiments (Fig. 2) and Radial 3 × 8 Target Task experiments performed either with a restrained-arm BMI or the hand (Fig. 3), we included all collected datasets where the monkey performed these tasks and was engaged in the task for the several hours that was typical for these animals. Several datasets were excluded because the animal chose to stop working unusually early that day (these had fewer than half as many trials as the included datasets). Eight monkey J datasets were analyzed (two were excluded due to insufficient trials). Ten monkey R datasets were analyzed (two were excluded due to insufficient trials). One additional monkey R dataset was excluded because the raw neural data recording system failed that day. The same dataset selection criterion was used for the free-arm BMI Radial 3 × 8 Target Task experiments (Fig. 4). Eight monkey J datasets were analyzed, and seven monkey R datasets were analyzed (two were excluded due to insufficient trials).

For the human clinical trial participant offline analyses (Fig. 5a–c), we analyzed three participant T5 and seven participant T6 BMI Grid Task datasets available to us from a prior study^2^; the methods section of that study describes its data collection protocol and block inclusion criteria. The closed-loop human participant decoder comparison (Fig. 5d,e) was only attempted during one research session, and this is the dataset that is reported.

### BMI decoders

The majority of experiments reported in this study examine neural activity while a BMI user moved a virtual cursor in a 2D workspace using a velocity Kalman filter. Before describing the several decoder variations used in this study, it is worth noting that as it is actually used in most BMI systems, including in the present work, the closed-loop operation of this class of decoders can be expressed^25,51,52^ in the form of:

1 $${\bf{v}}(t)={{\bf{M}}}_{1}{{\bf{v}}}_{{\rm{t}}-1}+{{\bf{M}}}_{{\bf{2}}}{{\bf{y}}}_{{\rm{t}}}$$

where **v**(t) = [v_hor_(t); v_ver_(t)] is the velocity vector the in horizontal and vertical workspace dimensions at a given time step t, **y**(t) is an E × 1 vector of neural features observed during the current time bin, E is the number of electrodes multiplied by the number of neural features per electrode, **M**_1_ and **M**_**2**_ are 2 × 2 and 2 × E matrices. This can be thought of as a second-order physical control system^18^ in which, at every time step, the velocity is a damped update of the previous velocity (in practice **M**_1_ is diagonal matrix with elements less than 1) plus a “neural push” input which is a linear weighting of neural activity (e.g., each electrode’s firing rates) onto horizontal and vertical velocity. The various ways to fit the decoder that are described below will adjust the specific parameters of **M**_1_ and **M**_2_ while leaving this essential form of the decoder the same.

An important deviation from this formulation is if the decoder has a Cursor Position Feedback subtraction operation to account for a potential influence of BMI cursor position on firing rates. Whether or not this operation is useful is one of the key questions of this study. This decoder operation is described in the next section.

In this study, BMI decoders were trained and used in the two different behavioral contexts: free-arm and restrained-arm. This entailed different protocols for obtaining the training data to fit the decoders. We followed the training protocols described in^19^ for the monkey BMI decoders and^2,24^ for the human BMI decoders, except where differences are noted. All decoders were fit and used within-context, by which we mean that decoders used in the free-arm context were trained from data collected during that context, and likewise, decoders used in the restrained-arm context were trained from passive observation or previous closed-loop BMI data collected during that context. The decoders operated with a 50 ms (monkeys) and 15 ms (humans) update time step, with velocity held constant for each 1 ms system update between neural updates.

In the monkey free-arm context and in T6’s ‘finger movements OK’ context, decoder training began with the subject/participant performing the Radial 8 Target Task with their hand. For two monkey J and one monkey R datasets, final decoders (with and without Cursor Position Subtraction) were fit from the initial hand-controlled block rather than from a first-pass decoder closed-loop block. This is explained more in the next section. For the remaining sessions, binned kinematics (hand endpoint velocity in the 2D vertical plane for monkeys, index and thumb velocities for participant T6) and neural data collected during this first hand-controlled block were used to fit an initial decoder. The subject/participant then used this initial decoder to perform another block of the Radial 8 Target Task. For participant T6, this recalibration block employed partial error attenuation to ensure that she could reach all targets^2^.

The closed-loop kinematics and neural data from this first-pass decoder block were used to fit a new, ‘recalibrated’ decoder. Previous work that introduced the ReFIT decoder, which includes a Cursor Position Subtraction operation (“Innovation 2” in^19^), tested the online benefit of this innovation by comparing ReFIT decoders with and without Cursor Position Subtraction while allowing the monkey to move his arm (their Supplementary Figures 3b and 4b). Both of these decoders included the ‘Cursor Goal’ intention estimation optimization (“Innovation 1’) of ReFIT, in which the training data velocities are rotated to point towards the target under the assumption that this is where the BMI user was always aiming. In the present study we also compared decoders with and without Cursor Position Subtraction (Fig. 1), also in the free-arm context, essentially replicating that aspect of the^19^ results. However, to provide more complementary new data, here we did not use the Cursor Goal innovation when training the decoders used for these comparisons. That is, our free-arm BMI closed-loop performance comparisons tested velocity Kalman filters fit from the same training data, without Cursor Goal correction, *either with or without Cursor Position Subtraction*.

In this study, Cursor Goal correction was used when fitting the decoders in the experiments comparing cursor positional effects during hand-controlled versus restrained-arm BMI task performance (Figs 2, 3) and in the human participant decoders (Fig. 5). It was not used in the free-arm BMI Radial 3 × 8 Target Task experiments (Fig. 4) in order to deliberately train sub-optimal decoders so as to reduce the correlation between hand and cursor positions.

During all decoder fittings, training data velocities were set to 0 during the target hold epoch (i.e., when the cursor was within the target acquisition area), as described in^19^. This operation is motivated by an assumption that neural activity reflects that the user is trying to command zero velocity during this epoch (even if some small movement may be unavoidable). Hold epoch velocities are therefore replaced with 0 velocity to more accurately fit the decoder to the user’s nominal intentions.

Decoder training during the restrained-arm BMI context was similar to the two-step free-arm protocol described above, in that an initial decoder was trained from an initial training block. This first decoder was then used to collect a closed-loop (BMI-controlled) second training data block, which was then used to train final decoders. The key difference, however, is that in the restrained-arm context, the initial training block consisted of the user watching automated straight-line movements of the cursor to the target, rather than performing the task with their hand. The decoder fitting procedure thus assumes that the neural activity being recorded corresponds to the kinematics of the (automated) cursor movement. While this need not be true, in practice this works well enough that a usable initial decoder can be fit and used to collect closed-loop data from which an improved (‘recalibrated’) final decoder can be fit.

When fitting the final decoders for the experiments comparing cursor positional effects during arm use and restrained-arm BMI use (Figs 2 and 3), two-thirds of the available monkey J electrodes and three-fourths of the available monkey R electrodes were used to causally control the cursor. A different random set of electrodes was chosen for decoder inclusion at the start of each experiment session (i.e., there was a different subset of electrodes used in each dataset). The original purpose of this experiment design choice was to potentially allow for comparing responses between electrodes that did or did not directly affect the decoder (‘direct’ or ‘indirect’ electrodes, respectively). However, initial analyses did not reveal substantial cursor positional effect differences between direct and indirect neural populations, and we did not further pursue this question in the present study; rather, all analyses combined both direct and indirect electrodes. The decoders used in the Fig. 4 experiments were also trained with only a subset of the electrodes. As mentioned earlier, this was to intentionally reduce the performance of these decoders. For monkey J, this fraction was one-half of the electrodes for six datasets, two-thirds for one dataset, and three-fourths for one dataset. For monkey R, three-fourths of the electrodes were used in all seven datasets.

The human participants had two-dimensional velocity control of a computer cursor, plus a discrete “click” selection command, using the ReFIT Kalman Filter and Hidden Markov Model-based state classifier detailed in^2^. Participant T5 attempted to move his (imagined to be) outstretched arm to command cursor velocity, whereas T6 attempted to move her index finger and thumb. Both participants “clicked” by attempting to squeeze their left (ipsilateral-to-arrays) hand. The human participants’ threshold crossing firing rates were smoothed online with a half-Gaussian kernel with 25 ms standard deviation. T5’s decoder operated only on threshold crossings, but T6’s decoder also operated on HLFP power because her array’s spikes signal quality was poor.

### BMI decoders with Cursor Position Subtraction

The hypothesis underling incorporating a Cursor Position Subtraction operation into a velocity decoder is that if the BMI effector’s position affects neural activity, then this positional effect can deleteriously affect BMI performance by causing changes in decoded velocity that are unrelated to the user’s actual movement intention. This can be mitigated by modeling the cursor positional effect as a nuisance variable and subtracting from the observed firing rates the expected contribution of the cursor’s current position.

In this study, we followed the method of^19^ and estimated the positional effect from neural and cursor position observations in the training data during time periods when the BMI user was holding the cursor over the target, right before target acquisition. The logic behind fitting Cursor Position Subtraction from the hold epoch is that we assume that during this period the user is engaged in the task and that therefore the sensorimotor system is tracking the position of the cursor. There is also less cursor movement during the hold period, which putatively reduces the amount of neural activity related to movement generation (e.g., intended speed and velocity tuning). This epoch therefore may better isolate cursor position-related activity.

Specifically, at the start of the decoder fitting procedure, let **Y** be the E × T matrix of the firing rate observations during all T target hold period time bins, and **P** = [**p**_hor_; **p**_ver_; **1**] is the 3 × T matrix of the mean cursor positions during each bin (the last bias row is to allow for baseline firing rates). We find **C**_pos_ = **Y**/**P**, where / is the matrix right division operation, i.e. the least-squares solution to **CP** = **Y**. The last column of **C**_pos_ is discarded, leaving an E × 2 matrix in which the *i*^th^ row provides weights describing how the horizontal and vertical cursor position are believed to affect the neural activity of electrode *i*.

**C**_pos_ is then used to subtract away **Y**_pos_ = **C**_pos_ * [**p**_hor_; **p**_ver_], i.e., the expected firing rate contribution due to the cursor’s position, from all neural observations during the subsequent steps of the Kalman filter fitting procedure and during online decoder operation. As a technical note, since this subtraction is applied during the fitting of the Kalman filter emissions matrix (the **C** matrix in the ReFIT decoder derivation from^19^), this is equivalent to fitting the emissions model of a position-velocity Kalman filter.

Thanks to the steady-state formulation of the velocity Kalman filter (equation 1), we can visualize the effect of the Cursor Position Subtraction operation as a vector field showing what ‘positional effect velocity’ is added to the decoded velocity just due to where the BMI cursor is located (Figs 1d, 5d). This contribution is calculated at a given position in the workspace as

2 $${{\rm{v}}}_{{\rm{pos}}{\rm{.effect}}}=-\,{{\bf{M}}}_{{\bf{2}}}{{\bf{C}}}_{{\rm{pos}}}[{{\rm{p}}}_{{\rm{hor}}}{;{\rm{p}}}_{{\rm{ver}}}]$$

where the negative sign reflects that the cursor positional effect is subtracted from observed firing rates.

As mentioned earlier, on three of the decoder comparison experiment sessions, the cursor positional effect was estimated from the monkey’s arm reaching data (data points marked with squares in Fig. 1c), rather than from closed-loop BMI data collecting using an initial decoder. Aside from this difference in how the training data was collected (i.e., whether the monkey was controlling the cursor with his hand or BMI), this cursor positional effect estimation procedure was the same as described above for when the effect was estimated from BMI task data. Our motivation for testing Cursor Position Subtraction decoders trained from both hand-controlled and brain-controlled data was to more comprehensively test when this operation improves performance in the free-arm BMI behavioral context.

The more important new closed-loop decoder comparisons were those collected in the restrained-arm BMI behavioral context. When we initially collected one monkey J dataset comparing the full ReFIT decoder (which includes Cursor Goal intention estimation) against ReFIT without Cursor Position Subtraction, we observed no online performance difference; both decoders performed very well. We speculated that perhaps the high performance on this task was close to a “ceiling effect”, thereby obscuring the benefit of Cursor Position Subtraction. We therefore collected the remaining monkey J datasets without the Cursor Goal operation during the decoder recalibration, which has previously been shown to reduce performance^19^. To further increase the range of online performance levels examined (i.e., to further reduce performance), three of these datasets had a randomly-chosen one-fourth, one-third, or one-half of electrodes disabled when fitting the final two decoders which were compared against one another (the same electrodes were removed for both decoders). Monkey R’s BMI performance was worse than monkey J’s, so we were less concerned about ceiling effects; we therefore compared the standard ReFIT decoder either with or without Cursor Position Subtraction in all seven of his restrained-arm datasets.

The same method for fitting and implementing a ReFIT decoder with Cursor Position Subtraction was used for the T5 closed-loop experiment collected specifically for this study.

### Cursor positional effect during target hold

These analyses seek to describe the relationship between neural activity and where in the workspace the BMI user is holding the cursor (Figs 2 and 5a–c). We divided the region of the workspace where targets could appear into either a 3 × 3 = 9 (monkeys J, R, and human participant T5) or 2 × 2 = 4 (participant T6) ‘tiles’ and grouped trials based on which tile the target was located in. We then trial-averaged hold-epoch firing rates across the trials. These choices of workspace division resolution were made to balance dividing the workspace into smaller regions to look for more nuanced position-dependent neural variability against the desire to have sufficient number of trials in each tile provide an accurate neural activity estimate. Across all of the datasets used in these ‘tile analyses’ (including both monkeys and human participants), the minimum number of trials within a tile was 18 trials, and the mean was 78.9 trials per tile).

The analyzed hold epoch for the monkey datasets was from 400 to 750 ms after the start of the target hold period (which lasted 800 ms). Firing rates were calculated by counting the number of spikes within these hold epochs and normalizing by its duration, yielding a single firing rate scalar per electrode per trial. For the human data analyses, we averaged each trial’s neural activity (firing rates for T5, HLFP power for T6) over the 100 ms before target selection (click). Cursor horizontal and vertical positions were averaged across this analysis epoch, yielding 2-element position vector for each trial. Non-functional electrodes (i.e., zero spikes recorded, or extreme noise) were excluded from these analyses.

We also reported across-tile neural modulation range as a percentage of movement epoch neural modulation range; this was done to provide a context for the “scale” of the cursor positional effect compared to the ‘dynamic range’ observed over the course of movements, which are known to elicit high activity in motor cortex. We trial-averaged firing rates in short time bins to capture the time-varying richness of peri-movement neural activity. Trial averaging, however, precluded using the Random Target and Grid Tasks in which each trial has a different start point, end point, and duration. Instead, this dynamic range was calculated by trial-averaging across the conditions of a Radial 8 Target Task block (approximately 200 trials) that was collected before each behavioral context’s Random Target (monkey) or Grid Task (human) blocks. Specifically, firing rates were computed from 0 to 600 ms after target on, using 20 ms bins slid by 1 ms; this provided sixteen condition-averaged (out to and back from each target) neural activity time series. For monkey datasets, this range was calculated by considering the minimum and maximum firing rates across both hand-controlled and restrained-arm BMI contexts.

We used a simple test to determine which dataset-electrodes to include in the Fig. 2d analysis: the electrode had to modulate during the movement task, which we defined as a firing rate change between the very start of the trial and a subsequent time window where the movement was being made. This test is motivated by the observation that the largest neural response component during arm movement tasks is a general change of firing rates when initiating a movement^53^. Specifically, for each electrode we binned spike counts in two windows during each trial (0 to 150 ms and 150 to 300 ms after target on) and compared these windows’ distributions; electrodes with a significant change (p < 0.01, sign-rank test) were considered ‘task-modulated’. This test was run separately for the monkey hand-controlled and restrained-arm BMI contexts and the electrode was included if it responded during either context. In practice, very few dataset-electrodes with significant cursor positional effects were excluded from analysis based on this movement tuning test.

The calculation of firing rate variance explained by cursor position reported in Fig. 2d was performed separately for each dataset-electrode, and consisted of a linear regression between **y**, individual trials’ firing rates during the analyzed target hold epoch (the same epoch as in the tile analysis) and the matrix of cursor positions on each trial [**p**_hor_; **p**_ver_; **1**]:

3 $${[\begin{array}{c}{{\rm{y}}}_{\mathrm{trial}1}\\ {{\rm{y}}}_{\mathrm{trial}2}\\ \ldots \\ {{\rm{y}}}_{\mathrm{trial}n}\end{array}]}^{T}=[\begin{array}{ccc}{\beta }_{1} & {\beta }_{2} & {\beta }_{3}\end{array}][\begin{array}{cccc}{{\rm{p}}}_{\mathrm{hor},\mathrm{trial}1} & {{\rm{p}}}_{\mathrm{hor},\mathrm{trial}2} & \ldots  & {{\rm{p}}}_{\mathrm{hor},\mathrm{trial}{\rm{n}}}\\ {{\rm{p}}}_{\mathrm{ver},\mathrm{trial}1} & {{\rm{p}}}_{\mathrm{ver},\mathrm{trial}2} & \ldots  & {{\rm{p}}}_{\mathrm{ver},\mathrm{trial}{\rm{n}}}\\ 1 & 1 & \ldots  & 1\end{array}]$$

where β_1_, β_2_, β_3_ are linear coefficients fit via least-squares regression. This formulation allows for a baseline firing rate for each electrode. The coefficient of determination (R^2^) of this regression was used to describe how well cursor position explained firing rate variance. To determine whether this variance explained was significant, we used a shuffle test in which we randomly permuted the pairings between trials’ cursor positions and firing rates and re-calculated the regression R^2^. This process was repeated over 1,001 shuffles. A p-value of below 0.001 for a given dataset-electrode indicates that trials’ true positions explained more of that dataset-electrodes’ firing rate variance (R^2^) than all the shuffled datasets’ cursor positions.

### Cursor positional effects during arm and BMI movements

The ‘workspace modulation’ metric used in this analysis (presented in Fig. 3) is described in the Results section. One additional detail is that since we were trial-averaging firing rates within a behavioral condition (BMI or arm use × target direction × workspace), we wanted to exclude any conditions with very few trials. This was to exclude firing rate measurements that were prone to unusually high single-trial noise. Specifically, we excluded any conditions with fewer than 10 trials, which ended up excluding 1 of 96 monkey J arm use conditions and 1 of 144 monkey R BMI use condition. To calculate the statistical significance of each dataset-electrode’s observed workspace modulation, we computed a null distribution of 1,001 shuffled workspace modulations. In each shuffle, workspace labels were randomly permuted amongst trials of a given behavioral context (arm or BMI use) and reach direction in that dataset. For example, during a shuffle an arm reach to the upward target in the left workspace might be re-labeled as an arm reach to the upward target in the right workspace. Each electrode’s workspace modulations were then re-computed when trial-averaging within the (shuffled) workspace groupings. This generates a distribution of workspace modulation magnitudes under the null hypothesis that firing rate differences are merely due to trial-to-trial variability that does not covary with which workspace the trial was actually performed in. The p-value of a given dataset-electrode’s statistic was determined based on how many of the shuffled workspace modulations its true workspace modulation was greater than.

### Cursor vs. hand positional effects during free-arm BMI use

Data from a Radial 3 × 8 Target Task where the monkey used a BMI with his arm free to move were analyzed to tease apart the separate neural correlates of hold-epoch cursor and hand positions (Fig. 4). As in the Fig. 2 analyses described in the previous section, we analyzed neural and kinematic data in an analysis epoch from 400 to 750 ms from the beginning of the target hold, prior to target selection. The monkey controlled the cursor solely with neural activity via the BMI; we did not enforce that his hand make any specific movements or that it be visible to the bead tracking system. Therefore, there were times when the bead was not tracked because the monkey turned his hand over or moved it to the extremes of his reach range (e.g., far away from or close to his body). Since we needed to measure the hand’s position during the analysis epoch for this analysis, we excluded trials in which the bead was not visible during the entire hold period. Across datasets, between 0% and 41.0% (mean 11.0%) of trials were excluded for this reason.

The firing rate variances explained by task axis cursor or hand positions (Fig. 4b) were calculated with linear regressions set up similarly to equation 3, except that only one position coordinate (horizontal or vertical, depending on the dataset’s task axis), plus the **1** for baseline firing rate, were predictor variables. Separate regressions were performed using either trials’ cursor or hand positions to try to predict these trials’ firing rates.

Partial R^2^ values (Fig. 4c) were calculated by squaring the linear partial correlation coefficients between trials’ hold-epoch firing rates and the task axis position of one effector (e.g., the hand) after controlling for that effector’s (e.g., the hand’s) covariance with the other effector (e.g., the cursor). For example, *ρ*_*neural, hand* · cursor_, the partial correlation between neural activity and hand position, after accounting for cursor position, was found by assembling all *n* trials’ hold-epoch firing rates into a length *n* vector, **y**. These trials’ hand and cursor positions were assembled into length *n* vectors **p**_hand_ and **p**_cursor_, respectively. MATLAB’s *partialcorr* (**y**, **p**_hand_, **p**_cursor_) function was then used to compute the partial correlation. *ρ*_*neural, cursor* · hand_, the partial correlation coefficient between firing rates and cursor positions, after controlling for hand positions, was found by interchanging hand and cursor in the above description.

Statistical significance for the partial correlations was calculated with a shuffle test similar to that described earlier for Fig. 2d: trials’ pairings between firing rates and kinematics (here, cursor and hand positions) were scrambled and partial R^2^ were calculated for each dataset-electrode from these 1,001 shuffled datasets. The p-value for a dataset-electrode’s partial R^2^ statistic was determined by how many of the scrambled datasets’ R^2^ its true data partial R^2^ was greater than.

## Data Availability

The data can be made available upon reasonable request by contacting the lead or senior authors.
