# Publisher Correction: Brain-machine interface cursor position only weakly affects monkey and human motor cortical activity in the absence of arm movements

**DOI:** 10.1038/s41598-018-37930-8

**Published:** 2019-03-28

**Authors:** Sergey D. Stavisky, Jonathan C. Kao, Paul Nuyujukian, Chethan Pandarinath, Christine Blabe, Stephen I. Ryu, Leigh R. Hochberg, Jaimie M. Henderson, Krishna V. Shenoy

**Affiliations:** 1 Neurosurgery Department, Stanford University, Stanford, CA USA; 2 Electrical Engineering Department, Stanford University, Stanford, CA USA; 3 Bioengineering Department, Stanford University, Stanford, CA USA; 4 Stanford Wu Tsai Neurosciences Institute, Stanford University, Stanford, CA USA; 5 Bio-X Program, Stanford University, Stanford, CA USA; 6 Neurobiology Department, Stanford University, Stanford, CA USA; 7 Howard Hughes Medical Institute at Stanford University, Stanford, CA USA; 8 Neurosurgery Department, Palo Alto Medical Foundation, Palo Alto, CA USA; 9 Center for Neurorestoration and Neurotechnology, Rehabilitation R&D Service, VA Medical Center, Providence, RI USA; 10 School of Engineering and Carney Institute for Brain Science Brown University, Providence, RI USA; 11 Department of Neurology, Harvard Medical School, Boston, MA USA; 12 Center for Neurotechnology and Neurorecovery, Department of Neurology, Massachusetts General Hospital, Boston, MA USA; 13 Electrical and Computer Engineering Department, University of California at Los Angeles, Los Angeles, CA USA

Correction to: *Scientific Reports* 10.1038/s41598-018-34711-1, published online 05 November 2018

In the original version of this Article, there were errors in Affiliations 4, 10 and 12 which were incorrectly listed as ‘Stanford Neuroscience Institute, Stanford University, Stanford, CA, USA’, ‘School of Engineering and Institute for Brain Science, Brown University, Providence, RI, USA’ and ‘Neurotechnology Trial Unit, Department of Neurology, Massachusetts General Hospital, Boston, MA, USA’ respectively.

The correct affiliations are listed below.

Affiliation 4

Stanford Wu Tsai Neurosciences Institute, Stanford University, Stanford, CA, USA

Affiliation 10

School of Engineering and Carney Institute for Brain Science Brown University, Providence, RI, USA

Affiliation 12

Center for Neurotechnology and Neurorecovery, Department of Neurology, Massachusetts General Hospital, Boston, MA, USA

These errors have now been corrected in the PDF and HTML versions of the Article.

